# Skeletal Muscle Microvascular Dysfunction Manifests Early in Diabetic Cardiomyopathy

**DOI:** 10.3389/fcvm.2021.715400

**Published:** 2021-07-20

**Authors:** Sadi Loai, Yu-Qing Zhou, Kyle D. W. Vollett, Hai-Ling Margaret Cheng

**Affiliations:** ^1^Institute of Biomedical Engineering, University of Toronto, Toronto, ON, Canada; ^2^Translational Biology and Engineering Program, Ted Rogers Centre for Heart Research, Toronto, ON, Canada; ^3^The Edward S. Rogers Sr. Department of Electrical and Computer Engineering, University of Toronto, Toronto, ON, Canada

**Keywords:** heart failure, preserved ejection fraction (HFpEF), microvascular dysfunction, ultrasound imaging, photoacoustic imaging, echocardiography

## Abstract

**Aim:** To perform a deep cardiac phenotyping of type II diabetes in a rat model, with the goal of gaining new insight into the temporality of microvascular dysfunction, cardiac dysfunction, and exercise intolerance at different stages of diabetes.

**Methods and Results:** Diabetes was reproduced using a non-obese, diet-based, low-dose streptozotocin model in male rats (29 diabetic, 11 control). Time-course monitoring over 10 months was performed using echocardiography, treadmill exercise, photoacoustic perfusion imaging in myocardial and leg skeletal muscle, flow-mediated dilation, blood panel, and histology. Diabetic rats maintained a normal weight throughout. At early times (4 months), a non-significant reduction (30%) emerged in skeletal muscle perfusion and in exercise tolerance. At the same time, diabetic rats had a normal, slightly lower ejection fraction (63 vs. 71% control, *p* < 0.01), grade 1 diastolic dysfunction (E/A = 1.1 vs. 1.5, isovolumetric relaxation time = 34 vs. 27 ms; *p* < 0.01), mild systolic dysfunction (ejection time = 69 vs. 57 ms, isovolumetric contraction time = 21 vs. 17 ms; *p* < 0.01), and slightly enlarged left ventricle (8.3 vs. 7.6 mm diastole; *p* < 0.01). Diastolic dysfunction entered grade 3 at Month 8 (E/A = 1.7 vs. 1.3, *p* < 0.05). Exercise tolerance remained low in diabetic rats, with running distance declining by 60%; in contrast, control rats ran 60% farther by Month 5 (*p* < 0.05) and always remained above baseline. Leg muscle perfusion remained low in diabetic rats, becoming significantly lower than control by Month 10 (33% SO_2_ vs. 57% SO_2_, *p* < 0.01). Myocardial perfusion remained normal throughout. Femoral arterial reactivity was normal, but baseline velocity was 25% lower than control (*p* < 0.05). High blood pressure appeared late in diabetes (8 months). Histology confirmed absence of interstitial fibrosis, cardiomyocyte hypertrophy, or microvascular rarefaction in the diabetic heart. Rarefaction was also absent in leg skeletal muscle.

**Conclusion:** Reduced skeletal muscle perfusion from microvascular dysfunction emerged early in diabetic rats, but myocardial perfusion remained normal throughout the study. At the same time, diabetic rats exhibited exercise intolerance and early cardiac dysfunction, in which changes related to heart failure with preserved ejection fraction (HFpEF) were seen. Importantly, skeletal muscle microvascular constriction

advanced significantly before the late appearance of hypertension. HFpEF phenotypes such as cardiac hypertrophy, fibrosis, and rarefaction, which are typically associated with hypertension, were absent over the 10 month time-course of diabetes-related heart failure.

## Introduction

Type II diabetes accounts for 90% of adult diabetes cases and is an increasingly prevalent condition worldwide, affecting 1 in 11 people (1). Diabetes has serious cardiovascular implications, as it carries a two to four times higher risk of heart failure (2) and the worst outcome (85% 5-year mortality) (3–5). People with diabetes can go on to develop heart failure with preserved ejection fraction (HFpEF), a difficult-to-diagnose phenotype because left ventricular contraction is normal. Unfortunately, many patients are undiagnosed as the condition progresses silently and slowly, and diagnosis occurs mostly late after heart damage has become irreversible, at which time few treatments remain as standard heart failure drugs fail. To enable earlier diagnosis for these patients, we need a greater holistic understanding of early abnormal changes both in the heart and outside the heart, and their relative temporal presentation.

In this study, we adopt a well-established rat model of type II diabetes to study the temporality of abnormal developments in cardiac function, microvascular function, and exercise capacity. While ample preclinical reports exist on diabetes, animal diabetes studies rarely assess long-term effects on the heart (6–8) and studies that do extend to cardiac function typically employ genetic models in which cardiac abnormalities manifest in a compressed time interval in young animals (9–13). As such, we lose the opportunity to discriminate the natural progression of a slowly developing disease and identify when along this timeline different structural and functional abnormalities first appear. We remedy this shortcoming by undertaking a time-resolved, longitudinal investigation of diabetic cardiomyopathy in rats, starting from 2 months old and continuing into mid-adulthood. A large array of diagnostic tools, both clinical and non-clinical, are deployed to monitor the evolution of cardiac and extra-cardiac changes. We hypothesize that measurable changes in microvascular function in skeletal muscle appear earlier than indicators of standard risk factors such as hypertension and elevated blood cholesterol. Our goal is to attain deep cardiac and microvascular phenotyping at different stages of diabetic cardiomyopathy.

## Materials and Methods

### Animals and Disease Induction

**Figure 1:**
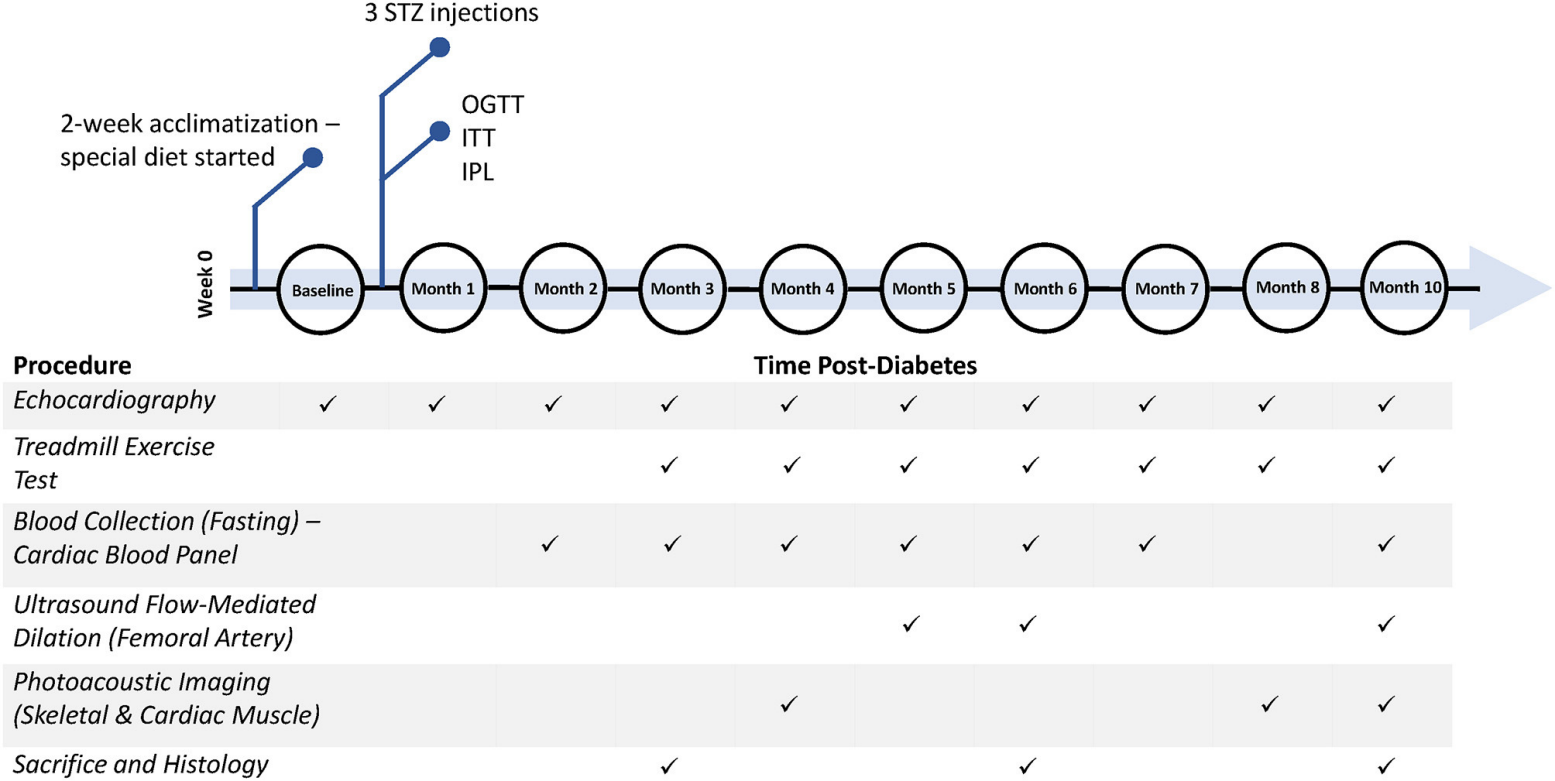
Timeline of diabetes induction and post-diabetes monitoring and assessment. Additional measurements not shown include weekly weight, weekly non-fasting blood glucose, and monthly blood pressure measurements. STZ, streptozotocin; OGTT, oral glucose tolerance test; ITT, insulin tolerance test; IPL, insulin plasma level.

This study was approved by our institutional Animal Care Committee (protocol #20012191), and all procedures were conducted in accordance with the Canadian Council on Animal Care. The full experimental timeline and all procedures performed are illustrated in Figure 1.

Six- to seven-week-old male Sprague Dawley rats (Charles River, Quebec, Canada) were housed two per cage with a 12:12-h light-dark cycle and constant room temperature (23 ± 1°C). Rats were divided into two groups: diabetic rats (*N* = 29) on a high-fat/high-sucrose diet containing 20.14 wt% sucrose and 20.68 wt% lard (D12451, Research Diets Inc., USA) and control rats (*N* = 11) on a calorie-matched control diet (D12450K, Research Diets Inc., USA). All rats were fed ad libitum on their respective special diet for the full duration of the study and had free access to water. After 2 weeks of acclimatization, diabetic rats were fasted overnight and injected intraperitoneally (IP) with a low dose of streptozotocin (STZ, 30 mg/kg, Millipore Sigma, 572201), dissolved in a 0.1 M citrate buffer, pH 4.5. The control group underwent the same procedure but were injected with a vehicle injection (citrate buffer). STZ injections were repeated a second time 2 weeks later in all 29 animals; 22 rats required a third injection, 2 weeks after the second injection, to chronically stress Langerhans islets cells and induce a state of frank diabetes. Diabetes was defined as having a non-fasting blood glucose level ≥ 15 mmol/L and a fasting blood glucose level ≥ 10 mmol/L. To ensure diabetes was maintained throughout the entire study, non-fasting blood glucose measurements (glucometer, Accu-Chek Aviva) from a tail vein prick were collected on a weekly basis.

### Confirmation of Glucose and Insulin Intolerance

Eight weeks after arrival, all rats underwent an oral glucose tolerance test (OGTT) to confirm glucose intolerance in diabetic rats. Animals were fasted overnight and then given an oral dose of glucose solution (BioShop Canada Inc.) at 1 g/kg *via* gavage. Blood glucose was measured over 2 h, prior to gavage and at 30-min intervals post-glucose consumption.

Nine weeks after arrival, all rats underwent an insulin tolerance test (ITT) to assess insulin resistance in diabetic rats. Animals were fasted for 6 h and injected IP with insulin solution (0.75 U/kg, Sigma-Aldrich, I9278). Blood glucose was measured prior to injection, at 15-min intervals post-injection for the first hour, and 30-min intervals for the second hour.

One-month post-diabetes (11 weeks after arrival), blood plasma insulin levels were tested in all rats to confirm insulin resistance in diabetic rats. Animals were fasted overnight, and blood from the saphenous vein was collected in lithium-heparin tubes. Tubes were centrifuged for 8 min at 8,000 rcf and blood plasma was separated from the samples. A rat insulin ELISA kit (Sigma-Aldrich, RAB0904) was used to determine insulin levels in the plasma samples. A homeostatic model assessment of insulin resistance (HOMA-IR) was then calculated as $\frac{Insulin\;\left(u\frac{IU}{mL}\right)\;x\;Fasting\;Glucose\;\left(\frac{mmol}{L}\right)\;}{22.5}\;$to assess insulin resistance (14).

### Cardiac Blood Panel

A cardiac blood panel was run at Months 2–7 and Month 10 post-diabetes to measure levels of high-density lipoprotein (HDL)-cholesterol, low-density lipoprotein (LDL)-cholesterol, total cholesterol, triglyceride, and fasting blood glucose. Animals were fasted overnight, and blood from the saphenous vein was collected in lithium-heparin tubes. Tubes were centrifuged for 8 min at 8,000 rcf and blood plasma was separated from the samples. Blood plasma was analyzed with a Beckman Coulter analyzer at The Centre for Phenogenomics.

### Blood Pressure Measurement

Non-invasive measurements of heart rate and systolic and diastolic mean blood pressure (BP) were taken monthly in conscious rats using a CODA Monitor (Kent Scientific, USA). Mean blood pressure was computed as $\frac{Systolic\;BP+\left(2*Diastolic\;BP\right)}{3}$. Rats were placed into holders and sufficiently warmed with a heating lamp for accurate BP readings. A cuff was then placed on the tail and the rat allowed to acclimatize to the restraint and cuff prior to BP measurement to reduce errors from motion. Blood pressure measurements were taken in a quiet environment with no sound or other disturbances.

### Treadmill Exercise Tolerance Testing

Exercise tolerance as determined by running distance on a treadmill was tested monthly, beginning at 3 months post-diabetes. All rats were placed on an exercise regime where the treadmill (Maze Engineers, USA) inclination was set at 12° and the speed started at a slow pace of 5 m/min for 2 min. The speed was increased to 10 m/min for another 2 min, followed by a 2 m/min increment every 2 min up to a maximum speed of 20 m/min or until the rat reached exhaustion. Exhaustion was defined as either the rat touching an electrical stimulus grid seven times with a minimum contact period of 1 s, or the rat failing to continue running within 7 s of full contact with the grid. The regime ended once the rat reached exhaustion or, in the absence of exhaustion, 20 min of exercise had elapsed. All rats were acclimatized to the treadmill regime for several days prior to data collection.

### *In-vivo* Ultrasound and Photoacoustic Imaging

A high-frequency ultrasound and photoacoustic imaging system (Vevo 3100/LAZR-X, FUJIFILM VisualSonics Inc., Toronto) equipped with transducers of different operating frequencies was used. Anesthesia was induced on 5% isoflurane (in 100% oxygen) delivered through a facemask for about 1 min and maintained on 1.5–2.0% isoflurane. The rat was then transferred to an imaging platform with temperature control, and the paws were taped to electrodes for electrocardiogram (ECG) signal monitoring. Body temperature was maintained at 37°C and monitored with a rectal thermal probe. The front chest and the hind leg were shaved, and remaining hair depilated with Nair cream for cardiac and leg imaging, respectively. Isoflurane was chosen among other anesthetics due to its fast-acting and short-lasting nature, and it is the preferred anesthetic for echocardiography for minimizing cardiorespiratory depression effects (15). Imaging sessions were kept under 20 min to avoid prolonged exposure to isoflurane and variability in heart rates between rodents.

#### Echocardiography

Transthoracic cardiac imaging was conducted monthly for all animals with a MS201 transducer (12–15 MHz). A protocol similar to that reported previously was followed (16). Two-dimensional B-mode imaging was used to visualize the LV inflow tract in a long-axis view; this view permitted clear demonstration of the pulmonary vein, left atrium, mitral orifice, and LV with clear borders of the epicardium and endocardium. M-mode recording was made at the middle segment of the LV through the largest chamber dimension. From the M-mode trace, the anterior and posterior LV wall thickness and the chamber dimensions at end systole and end diastole (ESD and EDD) were measured. The fractional shortening (FS%) and ejection fraction (EF%) were then calculated. Computed indices include LV end-diastolic volume (EDV) ($LVID_{d}^{3}*\frac{7}{2.4+LVID_{d}}$), LV end-systolic volume (ESV) ($LVID_{s}^{3}*\frac{7}{2.4+LVID_{s}}$), LV mass $\left(0.8*\left[{\left(LVID_{d}+LVPW_{d}+IVS_{d}\right)}^{3}-LVID_{d}^{3}\right]\right)$, and EF $\left(100*\frac{LV\;EDV-LV\;ESV}{LV\;EDV}\right),\;$where LVID represents the LV internal diameter at end systole and end diastole, LVPW_d_ represents the LV posterior wall thickness at end diastole, and IVS_d_ represents the inter-ventricular septum thickness at end diastole. In the long-axis view, pulmonary venous flow was located using color Doppler; pulsed-wave Doppler then recorded the blood flow velocity spectrum at the entrance of the pulmonary vein into the left atrium. Following a long-axis view, an apical four-chamber view was employed to record the left ventricular inflow spectrum at the mitral orifice using pulsed-wave Doppler. To ensure a strong, artifact-free blood flow signal, color Doppler was used to guide the placement of the pulsed-wave Doppler sample volume. Post-imaging analysis was performed to calculate standard parameters for assessment of LV function. In addition to measurements of LV structure (e.g., LV diameter), the mitral Doppler blood flow spectra was analyzed for the peak velocity of early diastolic wave (peak E), peak velocity of late diastolic wave (peak A), diastolic filling time (FT), systolic ejection time (ET), isovolumic relaxation time (IVRT), and isovolumic contraction time (IVCT) (17). Computed indices include the LV myocardial performance index (MPI) ($\frac{\left(IVRT+IVCT\right)}{Ejection\;Time}$), relative wall thickness (RWT) ($\frac{2*Posterior\;wall\;thickness}{LV\;Diastolic\;Diameter}$), and LV mass index ($\frac{LV\;mass\;\left(mg\right)}{body\;weight\;\left(g\right)}$). All data was analyzed over three to four consecutive cardiac cycles and an average of each parameter was taken.

#### Imaging of Femoral Arterial Flow-Mediated Dilation

Ultrasound imaging of FMD was conducted in the femoral artery, as described in Machin et al. (18). Briefly, rats were anesthetized as described above, the leg was shaved, and a 10 mm occlusion cuff (Holly Specialty Products, USA), inflatable with water, was placed on the ankle. Imaging was performed on the femoral artery, proximal to the cuff, using a MX400 transducer (30 MHz). Color Doppler was used to locate the femoral artery prior to pulsed-wave Doppler recording of the flow velocity spectrum at peak-systole and end-diastole. Data was collected at baseline and every minute during a 5-min occlusion period. After cuff release, data was collected every minute for three additional minutes. All data was analyzed over three to four consecutive cardiac cycles and an average of each parameter was taken. All animals recovered quickly after imaging, and no adverse effects resulted from the occlusion.

#### Photoacoustic Imaging of Myocardium and Skeletal Muscle

To estimate tissue perfusion, PA imaging of hemoglobin oxygen saturation (sO_2_) was performed at Month 4, 8, and 10 post-diabetes with a MX201 transducer for the myocardium and a MX400 transducer for leg skeletal muscle. The laser beam was calibrated with an external energy sensor prior to each imaging session to ensure measurements were normalized to the same energy scale. B-mode was used to locate the area of interest and to ensure that the targeted structure was within the laser beam's field-of-view. Myocardial PA imaging was conducted on the anterior LV wall in the long-axis view. Skeletal muscle PA imaging was performed beneath the femoral artery to avoid the large vessel during analysis. To ensure consistency at all timepoints, pre-determined gain level and other parameter settings were maintained for all animals. The average total sO_2_ (%) was calculated from each image over multiple cardiac cycles, with the region-of-interest kept at the same location for each animal during image analysis (19, 20).

### Histology

At the sacrificial timepoints indicated at Figure 1, rats were euthanized under 4% isoflurane and with 1 mL 10% potassium chloride injection into the right ventricle to stop the heart in end-diastole. The heart was perfused with saline and harvested. Tissues were fixed overnight in formalin and embedded in paraffin. The heart was sectioned in the transverse plane into three parts (atria, ventricles, and apex). Sectioned slices of the heart (5 μm) were stained with haematoxylin and eosin (H&E) for cross-sectional area (CSA), picrosirius red (PSR) for fibrosis, and CD31 (Abcam, EPR17259) for endothelial cell count. The leg skeletal muscle was divided into the gastrocnemius and gracilis muscles and stained with CD31. All sections were imaged on a Leica DMi8 inverted epifluorescence microscope using brightfield imaging. For each section, two to four slices, with five to ten images per slice covering all areas uniformly, were captured at 10X magnification. Myocardial and skeletal muscle capillary density was quantified in Python, utilizing OpenCV library for automatic shape detection and the Canny algorithm for edge detection, with consistent thresholding across all images. In assessing myocardial interstitial fibrosis, areas of periadventitial collagen surrounding vessels were avoided. Images were quantified for collagen content on ImageJ software, utilizing RGB stack images and consistent segmentation thresholds across all images (21). Cardiomyocyte CSA was assessed on ImageJ by measuring the individual CSA of 50–100 myocytes from the anterior and posterior LV wall.

### Statistical Analysis

All data plotted is represented as mean ± standard error of the mean (SEM). Data was analyzed using a two-way analysis of variance (ANOVA), where time and diet were independent variables, followed by a Fisher's *post-hoc* test for multiple comparisons in Matlab (version R2018a). Where only diet was a factor, a student's *t*-test was performed to determine significance. In all cases, significance was reported at a *p*-value of 5%.

## Results

### Diabetes Induction

**Figure 2:**
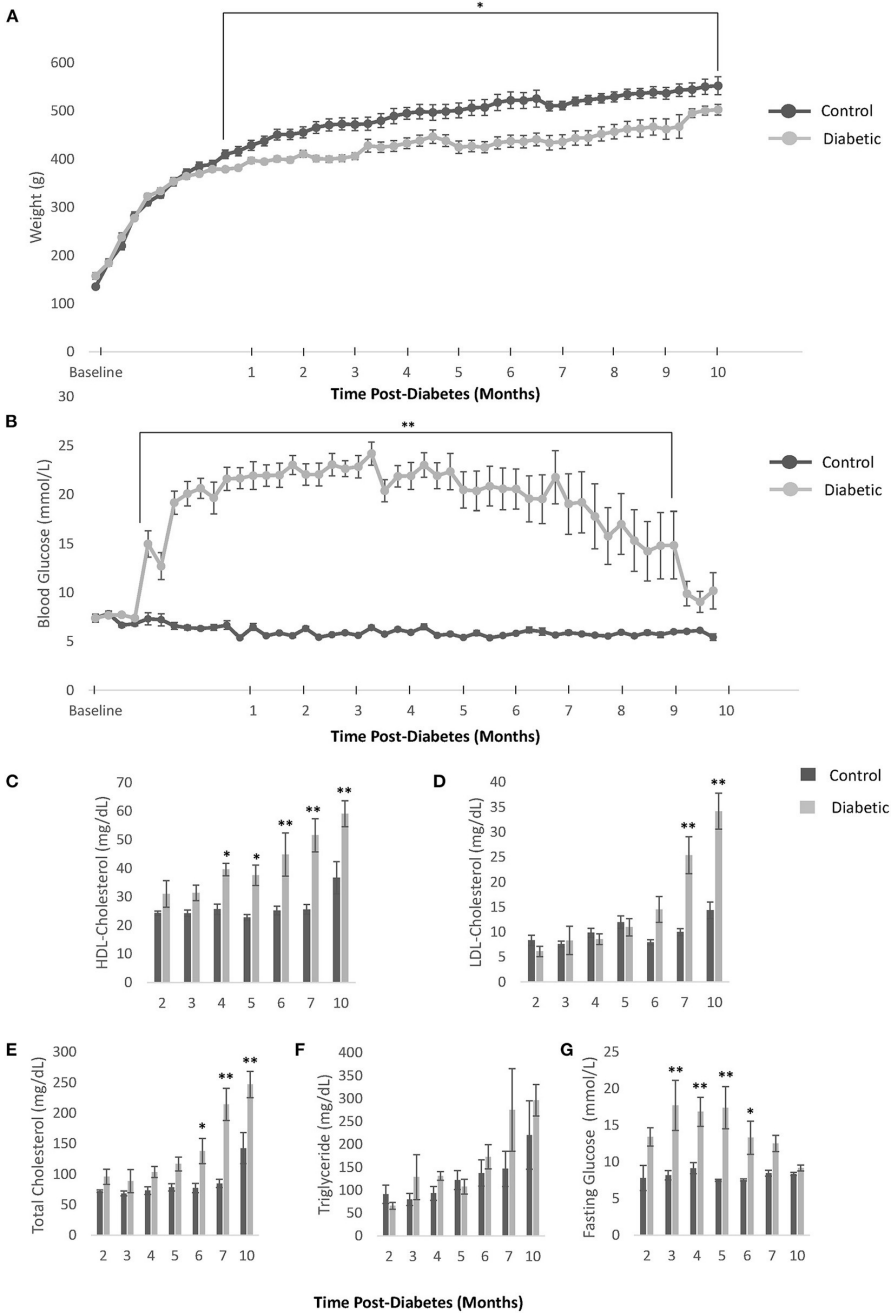
Body weight, blood glucose, and blood cholesterol levels. **(A)** Weekly weight measurements in all control and diabetic male rats. **(B)** Weekly non-fasting blood glucose measurements in all control and diabetic male rats. For **(C–G)**, data was obtained from *n* = 3 male control/diabetic at Month 2 post-diabetes; *n* = 5 male control/diabetic for Month 3–7; and *n* = 4 male control, *n* = 6 male diabetic at Month 10. **(C)** High-density lipoprotein (HDL) cholesterol over time. **(D)** Low-density lipoprotein (LDL) cholesterol over time. **(E)** Total cholesterol over time. **(F)** Triglyceride levels over time. **(G)** Fasting glucose blood levels over time. Significance at individual timepoints is indicated (*p < 0.05), (**p < 0.01). Data represented as mean ± SEM.

**Figure 3:**
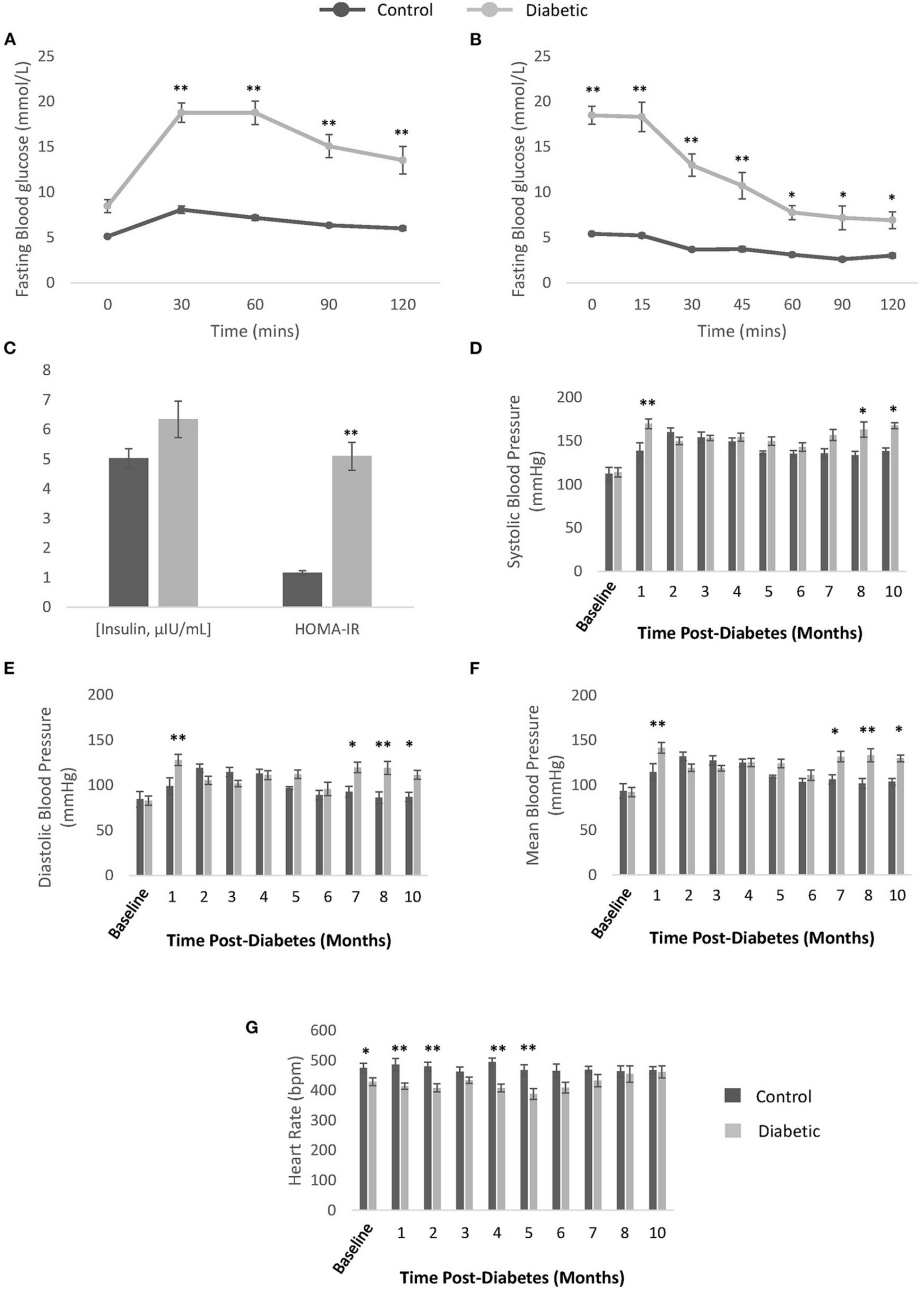
Confirmation of type II diabetes and non-invasive blood pressure measurements. **(A)** Oral glucose tolerance test performed after overnight fasting in control (*n* = 9) and diabetic (*n* = 19) rats. **(B)** Insulin tolerance testing performed after 6-h fasting in control (*n* = 9) and diabetic (*n* = 19) rats. **(C)** Blood insulin levels measured from plasma collected after overnight fasting (μIU/mL) and Homeostatic Model Assessment of Insulin Resistance (HOMA-IR) calculated for control (*n* = 7) and diabetic (*n* = 14) rats. **(D)** Systolic blood pressure. **(E)** Diastolic blood pressure. **(F)** Mean blood pressure. **(G)** Conscious heart rate [beats per minute (bpm)]. Significance at individual timepoints is indicated (*p < 0.05), (**p < 0.01). Data represented as mean ± SEM.

Our diabetes model did not induce obesity and led to the onset of hyperglycemia at Week 5, which remained significant beyond Month 9 post-diabetes (Figures 2A,B). Further confirmation of diabetes was obtained on OGTT, ITT, and blood insulin measurements to establish both glucose intolerance and insulin resistance (Figures 3A–C). The development of cataracts was noticed as early as 2 months post-diabetes in a few diabetic rats, but most of the diabetes cohort developed cataracts between 3 and 6 months post-diabetes, if at all. Cardiac blood panel measurements (Figures 2C–G) revealed late elevation of LDL-cholesterol, a presumed early indicator of diabetes (22), at Month 7. Likewise, elevated total cholesterol levels appeared late, and triglyceride levels were no different in the diabetic cohort relative to control. Unexpectedly, HDL-cholesterol was significantly increased early in the diabetes cohort from Month 4 onwards.

### Hypertension

Monthly conscious measurements of blood pressure and heart rate revealed a late elevation in blood pressure, from 8 months post-diabetes onwards (Figures 3D–G). The transient spike in blood pressure at 1 month is likely due to stress from multiple STZ injections and the fasting/blood collection procedures that occurred in the weeks prior. A slight depression in heart rate in the diabetes cohort was noted in the first 5 months post-diabetes. Note that the onset of hypertension coincides with significant reduction in skeletal muscle perfusion (see section Photoacoustic Imaging).

### Exercise Intolerance

**Figure 4:**
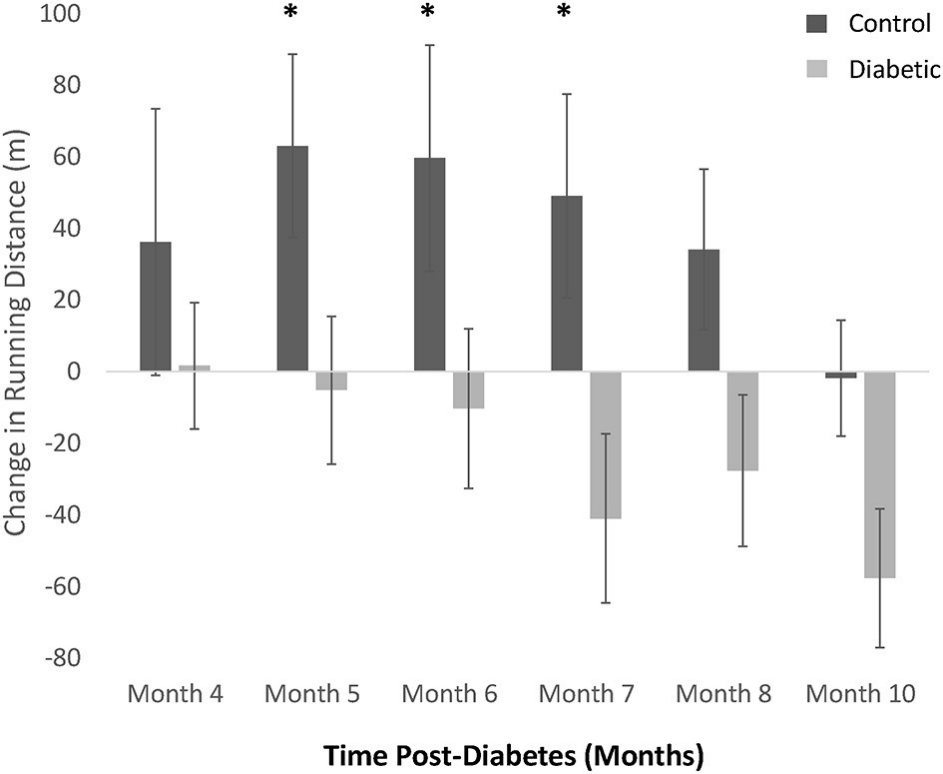
Treadmill exercise tolerance testing. The difference in distance run relative to Month 3 in individual rats is averaged within each cohort (control vs. diabetic). A significant difference between control and diabetic rats is observed at Months 5, 6, and 7. Significance at individual timepoints is indicated (*p < 0.05). Data represented as mean ± SEM.

Treadmill exercise testing revealed a continuing decline in running distance in the diabetes cohort, while the endurance of control rats continued to improve as they entered adulthood, peaking at Month 5, and thereafter declining toward but not reaching the running distance of diabetic rats (Figure 4). Due to individual differences in an animal's propensity to exercise, independent of disease, the change in running distance relative to Month 3 was calculated for each animal and averaged across the cohort. A significant discrepancy in exercise tolerance between control and diabetic rats appeared at Month 5, 6, and 7, when control rats were at their fittest.

### Echocardiography

**Figure 5:**
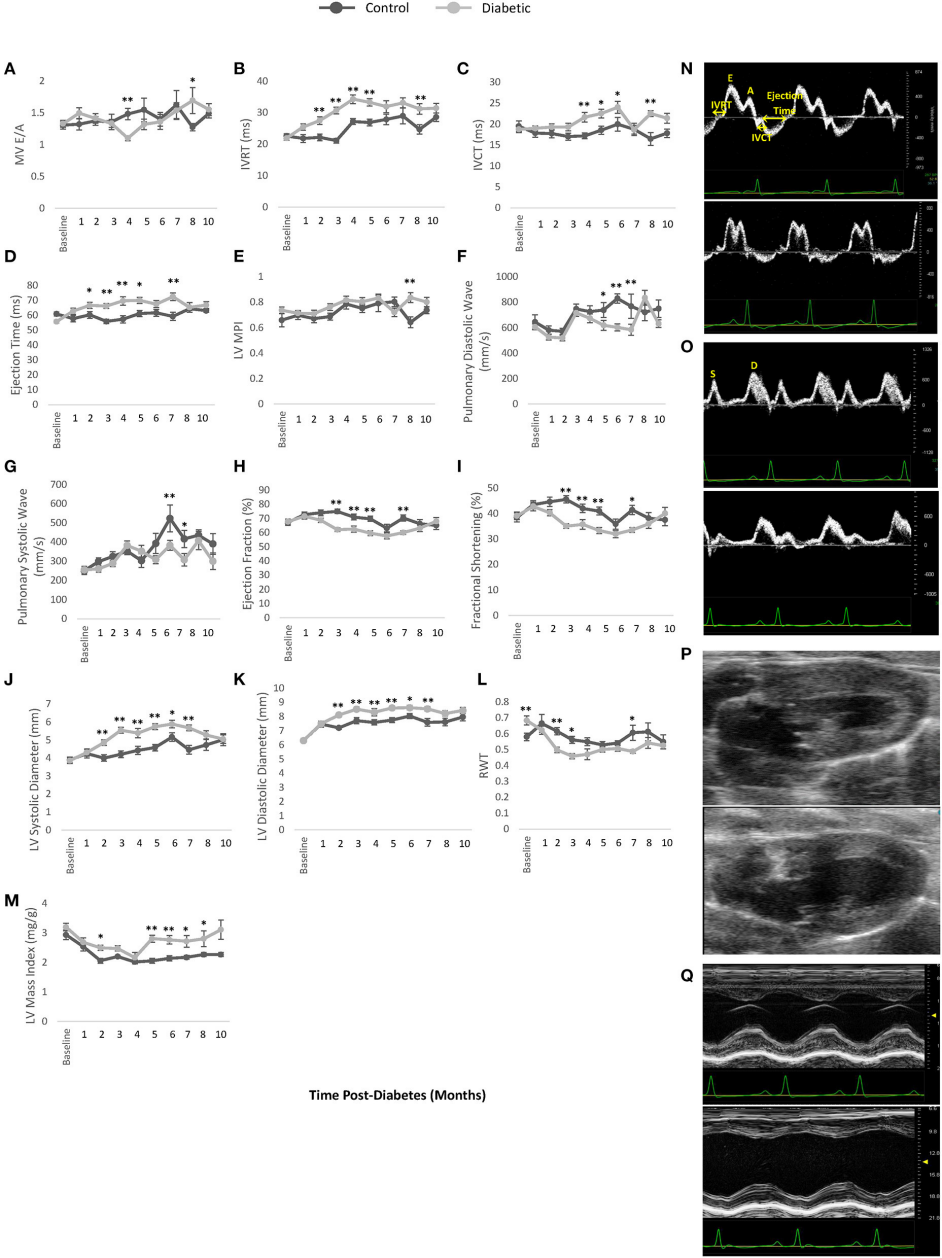
Cardiac LV function and dimensions measured by pulsed wave Doppler and M-mode echocardiography. **(A)** Mitral valve E/A ratio representing the ratio between peak blood velocity in the left ventricle (LV) during early diastole to peak blood velocity in the LV at late diastole. **(B)** Isovolumetric relaxation time (IVRT). **(C)** Isovolumetric contraction time (IVCT). **(D)** LV ejection time. **(E)** LV myocardial performance index (MPI). **(F)** Pulmonary peak diastolic (D) venous wave. **(G)** Pulmonary peak systolic (S) venous wave. **(H)** Left ventricle (LV) ejection fraction. **(I)** Fractional shortening. **(J)** LV diameter at end-systole. **(K)** LV diameter at end-diastole. **(L)** Relative wall thickness (RWT). **(M)** LV mass index. **(N)** Pulsed wave Doppler ultrasound images taken at the mitral orifice in the apical four-chamber view at 4 months post-diabetes for male control (top) and diabetic (bottom) rats. **(O)** Pulsed wave Doppler ultrasound images taken at the entrance of the pulmonary vein into the left atrium at 6 months post-diabetes for male control (top) and diabetic (bottom) rats, highlighting the pulmonary peak systolic (S) and diastolic (D) venous wave. **(P)** B-mode ultrasound images taken of the LV at end-systole in the long-axis view at 3 months post-diabetes for male control (top) and diabetic (bottom) rats. **(Q)** M-mode ultrasound images taken at the middle segment of the LV through the largest chamber dimension in the long-axis view at 3 months post-diabetes for male control (top) and diabetic (bottom) rats. Significance at individual timepoints is indicated (*p < 0.05), (**p < 0.01). Data represented as mean ± SEM.

**Figure 6:**
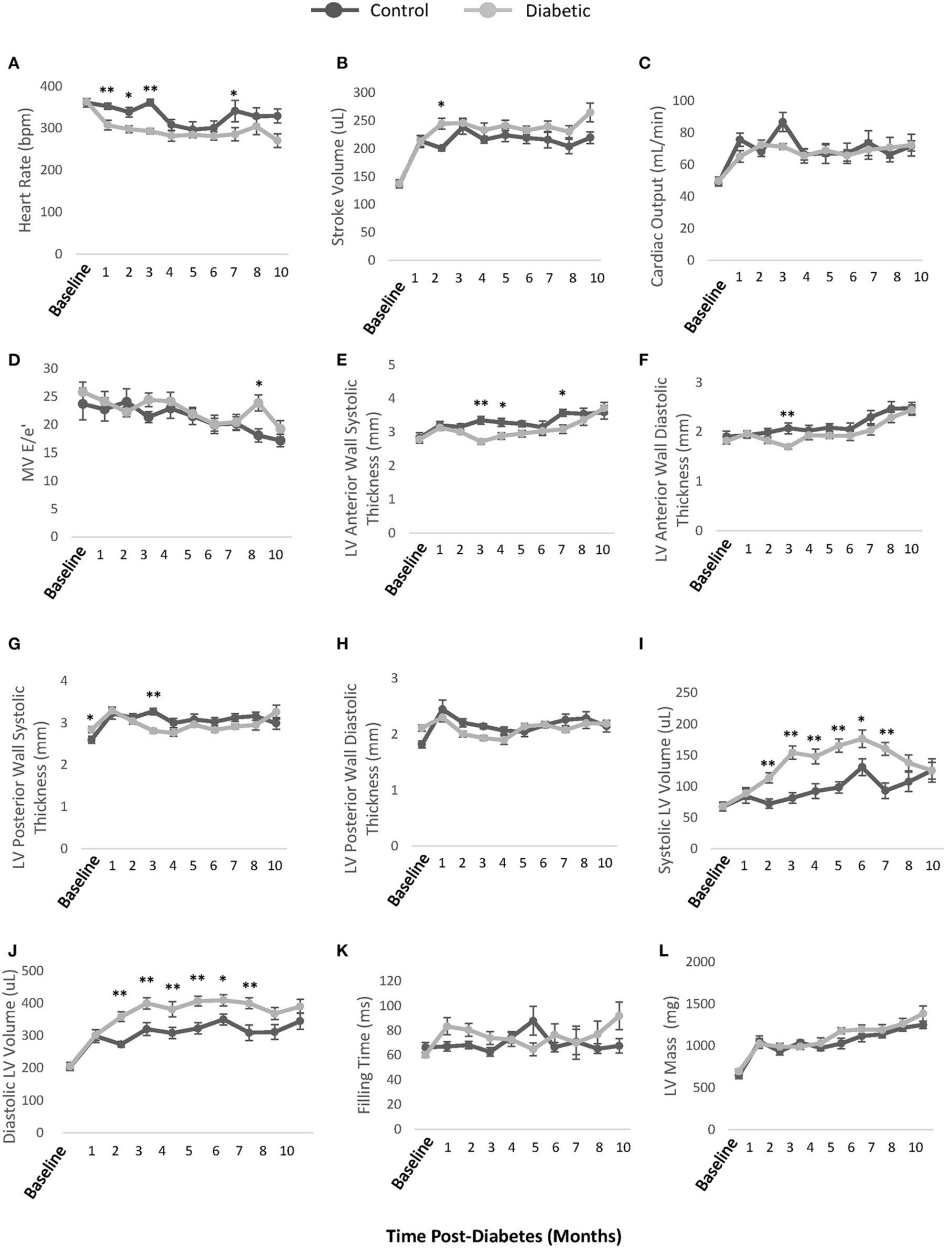
Additional echocardiographic parameters. **(A)** Unconscious heart rate [beats per minute (bpm)]. **(B)** LV stroke volume calculated as EDV-ESV. **(C)** LV cardiac output calculated as heart rate × stroke volume. **(D)** Mitral valve E/e' ratio representing atrial filling pressure, defined as the ratio between peak blood velocity in the LV and peak mitral annular velocity during early diastole. **(E)** LV anterior wall thickness at end-systole. **(F)** LV anterior wall thickness at end-diastole **(G)** LV posterior wall thickness at end-systole. **(H)** LV posterior wall thickness at end-diastole. **(I)** LV volume at end-systole. **(J)** LV volume at end-diastole. **(K)** LV Filling time. **(L)** LV mass. EDV, end-diastolic volume; ESV, end-systolic volume. Significance at individual timepoints is indicated (*p < 0.05), (**p < 0.01). Data represented as mean ± SEM.

Monthly echocardiography assessment provided a temporally resolved depiction of structural and functional changes in the heart and age-matched comparisons between control and diabetic rats (Figure 5). Diastolic dysfunction was observed in the diabetes cohort, as revealed in a significantly lower E/A ratio (grade 1 diastolic dysfunction with abnormal relaxation) at Month 4 post-diabetes and a significantly higher E/A ratio (grade 3 diastolic dysfunction with reversible restrictive filling) at Month 8 after an interval of pseudo-normalization (Figure 5A). Note that an evolving E/A ratio is the reason why it is extremely difficult to identify diastolic dysfunction when echocardiographic assessments are not performed longitudinally (23). The IVRT, another parameter used clinically to determine diastolic dysfunction, was elevated in diabetic rats from Month 2 onwards (Figure 5B). However, mild systolic dysfunction was also present, as evidenced in elevated IVCT and ejection times in diabetic rats (Figures 5C,D). Two less commonly used parameters corroborated the presence of both diastolic and systolic dysfunction: a depressed pulmonary vein diastolic wave and an elevated LV MPI, respectively (Figures 5E,F). Despite these functional changes, the LV ejection fraction was preserved, although values were slightly lower in diabetes (Figure 5H). Fractional shortening was also lower (Figure 5I). Structurally, the diabetic heart was enlarged with a slightly reduced LV wall thickness (RWT) but an overall increase in LV mass index (Figures 5J–M). Over a dozen other echocardiographic parameters are summarized in Figure 6.

### Femoral Arterial Flow-Mediated Dilation

**Figure 7:**
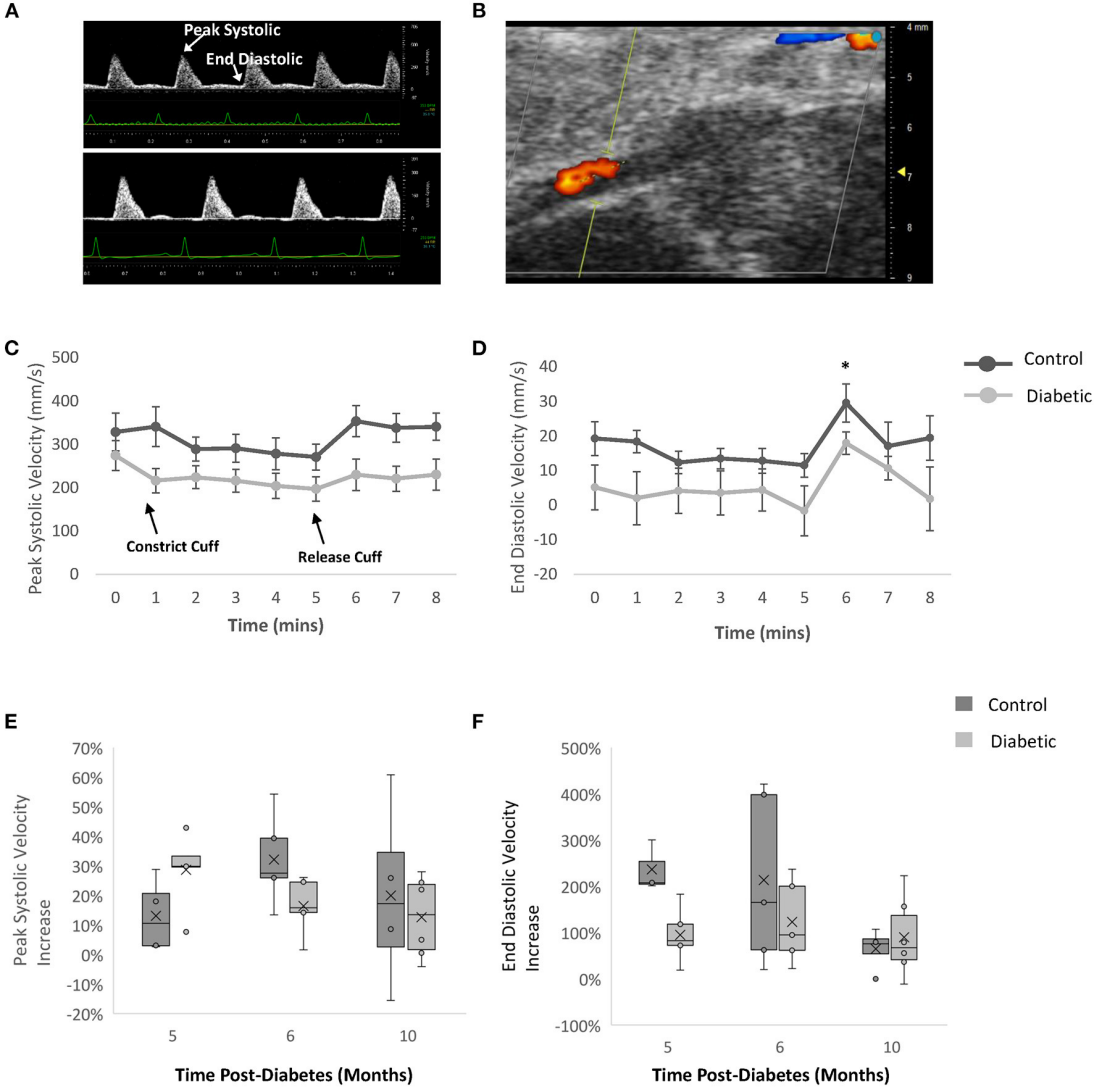
Flow-mediated dilation measurements of femoral artery by Doppler ultrasound. Blood velocity measured via color Doppler with a cuff on the ankle at Month 5, 6, and 10 post-diabetes. **(A)** Femoral artery Doppler ultrasound images at Month 6 - baseline for male control (top) and diabetic (bottom) rats. **(B)** Representative femoral artery color Doppler ultrasound image at Month 6 – baseline. **(C)** Peak systolic velocity and **(D)** end diastolic velocity at Month 6 post-diabetes measured in control (*n* = 5) and diabetic (*n* = 5) rats. Significance was evaluated between pre-cuffing (5 min) and cuff release (6 min), and difference was observed for both control and diabetic cohorts. Relative change in **(E)** peak systolic velocity and **(F)** end diastolic velocity post-cuff release, measured at Month 5 (*n* = 5 control, *n* = 5 diabetic), Month 6 (*n* = 5 control, *n* = 5 diabetic), and Month 10 (*n* = 4 control, *n* = 6 diabetic) post-diabetes. Line graphs represented as mean ± SEM. Box plot mean value is represented with the symbol “X.” Significance at individual timepoints is indicated (*p < 0.05).

FMD measurements on the femoral artery revealed a significant increase in blood flow upon cuff release in both the control and diabetic cohort, but the percentage increase was not different between the two cohorts (Figure 7). This result demonstrates that the reactivity of the femoral artery was normal, as expected. However, we noted a significantly lower baseline blood velocity in diabetic rats, most likely attributed to reduced microvascular perfusion in leg muscle (see section Photoacoustic Imaging).

### Photoacoustic Imaging

**Figure 8:**
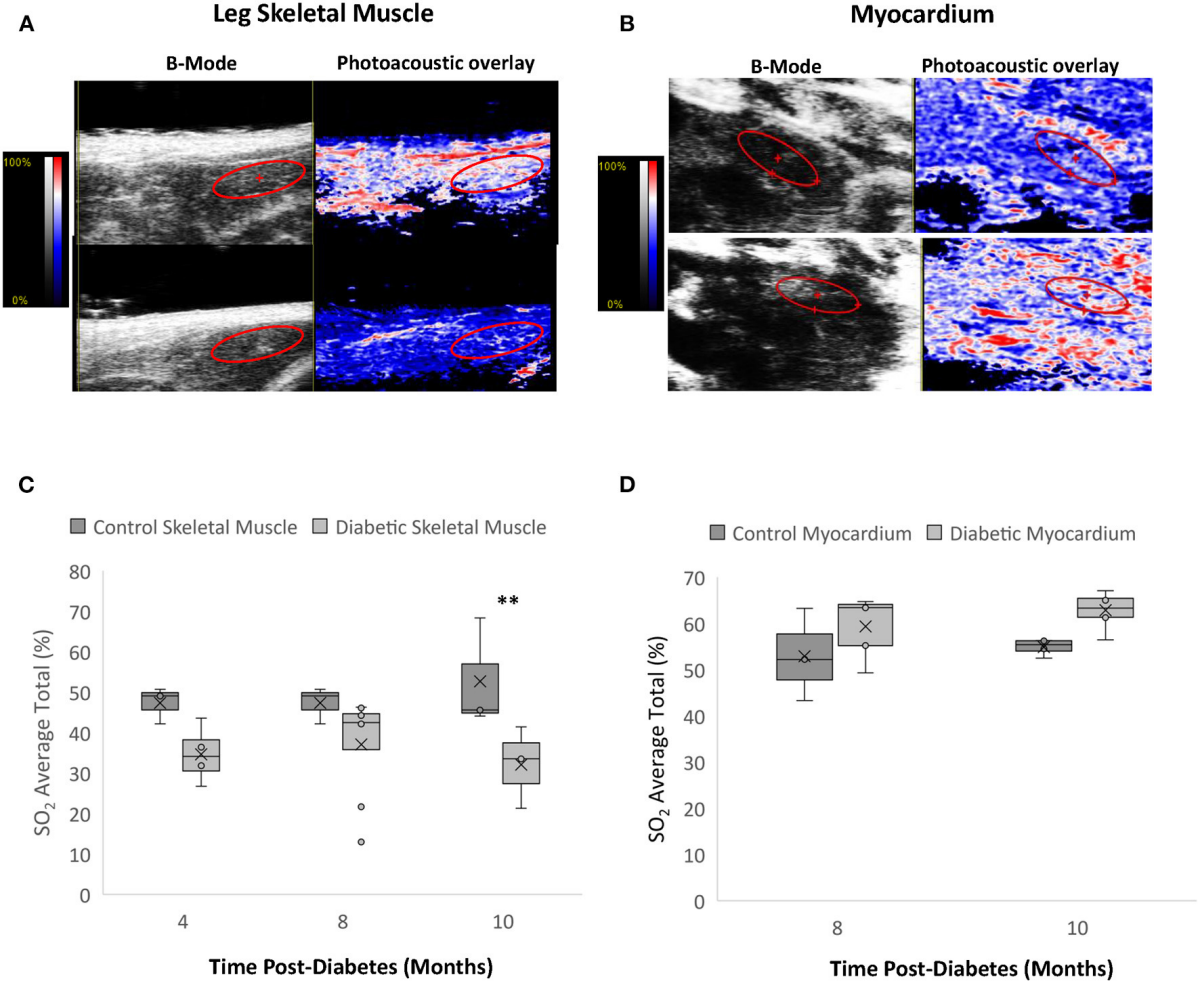
Photoacoustic imaging in leg skeletal muscle and myocardium. **(A)** Photoacoustic ultrasound images of male control (top) and diabetic (bottom) skeletal muscle at 10 months post-diabetes. Greyscale images are B-Mode images, and colormaps are the photoacoustic blood oxygenation saturation overlay. **(B)** Photoacoustic ultrasound images of male control (top) and diabetic (bottom) cardiac muscle at 10 months post-diabetes. **(C)** Blood oxygen saturation average in skeletal muscle at 4 months (*n* = 3 control and *n* = 4 diabetic), 8 months (*n* = 3 control and *n* = 8 diabetic), and 10 months (*n* = 4 control and *n* = 6 diabetic) post-diabetes. **(D)** Blood oxygen saturation average in the myocardium at 8 months (*n* = 3 control and *n* = 5 diabetic) and 10 months (*n* = 4 control and *n* = 6 diabetic) post-diabetes. Box plot mean value is represented with the symbol “X.” Significance at individual timepoints is indicated (**p < 0.01).

Photoacoustic imaging of blood sO_2_ revealed significantly reduced leg skeletal muscle perfusion but mildly elevated myocardial perfusion in diabetic rats (Figure 8). In skeletal muscle, reduced perfusion was seen at the first measurement timepoint of Month 4, with significance emerging at Month 10 post-diabetes. While the change in the myocardial perfusion never reached significance, a similar observation of higher sO_2_ levels was noted in type II diabetic patients with elevated glycohemoglobin A1c levels (24).

### Histology

**Figure 9:**
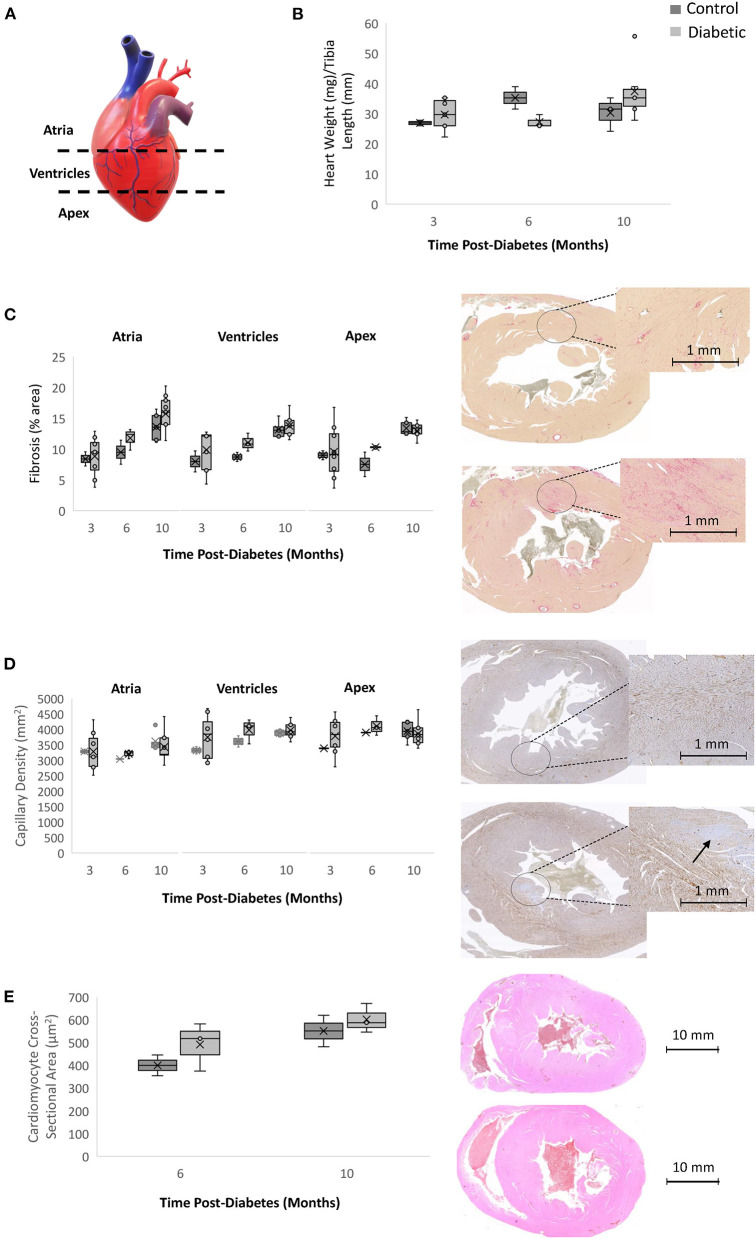
Histology analysis on rodent hearts. Timepoints refer to time post-diabetes at 3 months (*n* = 2 control, *n* = 8 diabetic), 6 months (*n* = 2 control, *n* = 3 diabetic), and 10 months (*n* = 5 control, *n* = 9 diabetic). **(A)** Schematic representing the three sections of the heart where histology was performed (atria, ventricles, and apex). **(B)** Heart weight measured immediately post-sacrifice and heart weight-to-tibial length ratio. **(C)** Interstitial fibrosis quantified as percentage of collagen surface area coverage on stained heart sections; shown on the right are slide scans of the LV at 10 months post-diabetes in control (top) and diabetic (bottom) rats. **(D)** Myocardial capillary density quantified as the number of vessels per mm^2^ of tissue on stained heart sections; shown on the right are slide scans of the LV at 10 months post-diabetes in control (top) and diabetic (bottom) rats. Arrow shows a small area of reduced capillary density. **(E)** Cardiomyocyte cross-sectional area quantified at 6 months (*n* = 2 control, *n* = 3 diabetic) and 10 months post-diabetes (*n* = 2 control, *n* = 3 diabetic); shown on the right are H&E slide scans at 10 months in control (top) and diabetic (bottom) rodent hearts. Box plot mean value is represented with the symbol “X”.

Histological analysis for fibrosis and microvascular rarefaction, two commonly assessed pathologies in heart failure, was completed for three sections of the heart (atria, ventricles, and apex) (Figure 9). There was a trend toward increasing interstitial fibrosis with age in both the control and diabetes cohorts, but no difference emerged overall despite the appearance of clusters of darker stain at Month 10 in diabetic rats. Myocardial capillary density count was also not different between the diabetic and control cohorts, indicating the absence of significant rarefaction in the diabetic heart. Lastly, cardiomyocytes were slightly larger in the diabetic heart, but the difference did not reach significance.

**Figure 10:**
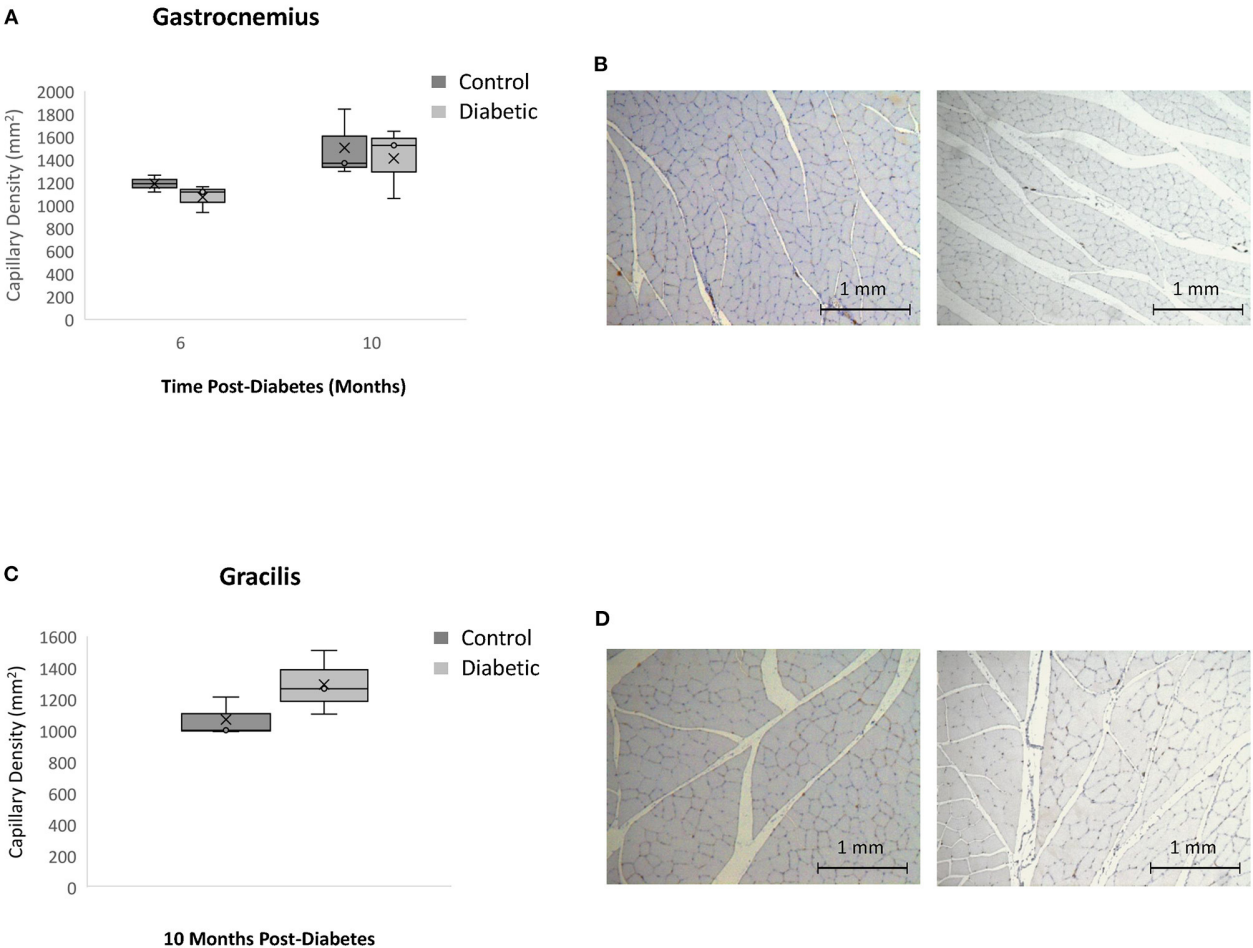
Histology assessment of skeletal muscle capillary density. Timepoints refer to time post-diabetes at 6 months (*n* = 2 control, *n* = 3 diabetic) and 10 months (*n* = 3 control, *n* = 3 diabetic). **(A)** Gastrocnemius muscle capillary density quantified as the number of vessels per mm^2^ of tissue on CD31 stained sections. **(B)** Gastrocnemius sections stained with CD31 of control (left) and diabetic (right) rats at 10 months post-diabetes. **(C)** Gracilis muscle capillary density quantified as the number of vessels per mm^2^ of tissue on CD31 stained sections. **(D)** Gracilis sections stained with CD31 of control (left) and diabetic (right) rats at 10 months post-diabetes. Box plot mean value is represented with the symbol “X”.

In skeletal muscle, specifically the gastrocnemius and gracilis muscles, there was no significant difference in capillary density between diabetic and control animals at Month 10 (Figure 10). This result rules out microvascular rarefaction as the underlying source for reduced leg perfusion seen on photoacoustic imaging. Barring structural abnormalities, reduced skeletal muscle perfusion can only arise from functional anomalies, such as a vasoconstrictive state.

## Discussion

Using a well-validated diet-induced, low-dose STZ model of type II diabetes in rats, this 10-month longitudinal study uncovered new information on the sequence of pathological changes preceding and coinciding with the development of diabetic cardiomyopathy, with multiple overlaps with HFpEF characteristics. In the following, we shall examine these changes in detail one by one. Overall, we observed early a reduction in leg skeletal muscle perfusion and lower exercise tolerance in diabetic subjects. At the same time, the heart began to undergo pathological changes while maintaining a preserved ejection fraction.

Exercise intolerance is one of the key symptoms of heart failure in general. However, the temporal relationship between changes in exercise tolerance and disease progression has not been disentangled (25). Our study revealed that in the absence of obesity, diabetic rats started to exhibit diminished exercise capacity as early as 4 months post-diabetes. While control rats continued to see an increase in exercise tolerance as they matured into adulthood, the running distance of the diabetes cohort stagnated or decreased further with time. One possible explanation for the exercise intolerance seen consistently in diabetic animals is reduced skeletal muscle perfusion of the legs. Photoacoustic imaging confirmed that diabetic rats had a reduced sO_2_ as early as 4 months post-diabetes. When this perfusion abnormality is taken together with the results of FMD measurements, which demonstrated normal femoral arterial reactivity but a depressed baseline blood flow, we can conclude that the skeletal muscle microvascular bed in diabetic animals shifted to a vasoconstrictive state in the absence of rarefaction.

The evolving structural and functional changes of the heart were also captured on echocardiography with unprecedented temporal resolution in this study. A preserved ejection fraction and normal cardiac output were maintained throughout, and a diagnosis of diastolic dysfunction was confirmed on measurements of the E/A ratio, IVRT, and pulmonary diastolic wave. Changes in E/A appeared early at Month 4, indicating the onset of early (grade 1) diastolic dysfunction at the same time as manifestation of exercise intolerance and reduced skeletal muscle perfusion. Incidentally, the E/e' ratio, a marker commonly used in the clinic to diagnose increased LV filling pressure, was elevated only at Month 8. Structural changes were also visible. As early as 2 months post-diabetes, the LV diameter and volume had increased in the diabetes cohort and the LV wall had begun to thin slightly. Although the LV chamber of control rats also enlarged with age, that of diabetic rats was consistently larger. It is important to emphasize that this type of cardiac remodeling is consistent with a broad array of human HFpEF samples, which have established many patterns of remodeling different from the archetypical hypertrophic, small-chambered heart reported in early HFpEF studies (26, 27). Furthermore, both concentric and eccentric hypertrophy have been observed in clinical HFpEF cases (28–30). Lastly, although ejection fraction is preserved, systolic function is not necessarily normal (26). Our diabetic rats exhibited slightly elevated IVCT and ejection times consistent with mild systolic dysfunction.

Histologically, we did not observe significant interstitial fibrosis in the myocardium. While this agrees with some literature studies of HFpEF in rodents and porcine models (31, 32), we also recognize that fibrosis, if significant, has proven not adequately specific for HFpEF in patients (33, 34). HFpEF is a heterogeneous disease, and the presence of cardiac fibrosis may be related to specific comorbidities whose association remain to be elucidated. What is clear from our data, however, is that both the healthy and diabetic heart are associated with an increase in collagen content with age, and the appearance of small patches of diffuse fibrosis on slide scans at Month 10 in diabetic rats corroborate fibrosis as a late-stage marker after hypertension had already set in. Assessment of myocardial microvascular density revealed no rarefaction was present in diabetic rats. Slide scans uncovered small regions of lower endothelial cell count. Rarefaction was also absent in leg skeletal muscle. Contrary to findings in the heart, however, perfusion was significantly lower in skeletal muscle of diabetic animals – an integrated interpretation of histological and photoacoustics data points to the early presence of functional, but not structural, microvascular abnormalities in skeletal muscle in diabetes.

Blood analysis also shed light on the utility of clinical blood biomarkers. Several biomarkers widely used in the clinic for predicting heart disease risks (e.g., elevated LDL-cholesterol, total cholesterol, and triglycerides) presented in later stages, several months after structural and functional cardiac changes manifested. On the other hand, HDL-cholesterol, which is not commonly associated with risks, was significantly elevated starting at Month 4 post-diabetes. While this result was unexpected, elevated levels of HDL have been reported previously, specifically in diseases where systemic inflammation played a major role and ultimately led to endothelial and renal dysfunction (35, 36). Interestingly, non-fasting blood glucose level began to decline at Month 9. While this was an unexpected finding, similar declines in blood glucose have been reported at the same time interval in rats with type II diabetes (7). Recovery of insulin production was eliminated as an initiator, as continued limited insulin production was confirmed at the end of the study (7). Weight loss as a reason for improved blood glucose is also implausible, as our diabetic animals maintained their weight until the end of the study. What caused a yet unknown biological switch to act in synchrony across our study and theirs warrants further investigation.

It is important to note that a non-obese diabetes model was employed in this study to eliminate the influence of obesity. The two diets given to diabetic and control rats were matched in calories; also, 45% of calories (as opposed to the more common 60%) in the high-fat/high-sucrose diet was derived from fat. This diet resulted in non-obese diabetic rats. In fact, diabetic rats weighed slightly less than control animals throughout the study, a difference that can be attributed to diabetic rats having more frequent urination (cages were changed twice to three times instead of once weekly), drinking more water to maintain hydration, and consuming slightly less food. Dehydration and lower food consumption, when compounded with potential muscle atrophy from inactivity, would account for a slightly lower body weight in diabetic animals.

Interpreting all phenotyping results collectively, we arrive at several important conclusions in relation to the temporal evolution of disease in diabetic cardiomyopathy. First, microvascular dysfunction presented early in the skeletal muscle of the legs in the form of vasoconstriction, even though quantitatively the measurements were not drastically different until months later. This observation is not surprising, given the known early involvement of the microvasculature in diabetes. What was unexpected was the absence of microvascular dysfunction in the heart muscle, at least from the lens of altered baseline perfusion or vessel rarefaction. A second conclusion is that exercise tolerance declined early at around the same time as leg muscle microvascular dysfunction. While lower exercise capacity may be partly attributed to early changes in the heart, preservation of ejection fraction (in addition to preserved cardiac output, stroke volume, and myocardial performance index) suggests that leg perfusion deficits played a larger role in diminished exercise capacity. A third conclusion is the absence of macrovascular dysfunction during the time course of this study, as FMD measurements of the femoral artery demonstrated normal reactivity. Again, this observation is consistent with our knowledge that vascular dysfunction first manifests in the microvasculature and later in larger vessels; abnormalities in FMD assessment may emerge much later. A fourth conclusion is that hypertension and elevated blood cholesterol, both considered standard risk factors for heart disease, both appeared late in diabetes and well after leg muscle microvascular dysfunction was first observed. Given that these metrics are routinely used for assessing risk, it may be beneficial to incorporate earlier stage markers, such as muscle perfusion, to improve diagnosis and prognosis. The fifth and final main conclusion is that even when heart function was compromised, as evidenced by mild systolic dysfunction and late-stage diastolic dysfunction, the classical signs of cardiac hypertrophy, fibrosis, and myocardial rarefaction were absent. Perhaps given sufficient time after the late onset of hypertension in diabetes, these more striking “classical” features will also emerge.

Several limitations in this study deserve mention. An invasive pressure-volume analysis was not conducted to evaluate elevated LV filling pressure. Stress testing was also omitted but may offer insight into early functional changes. Female animals were not included. The number of control animals for histology assessment at months 3 and 6 can also be boosted despite low variation in measurements. Finally, a more protracted timeline was precluded based on considerations for the welfare of animals subjected to this disease model; had we been able to continue the study after the late onset of hypertension, it is likely late-stage heart failure phenotypes would appear, including myocardial fibrosis, microvascular rarefaction, and myocardial thickening.

In conclusion, we have described a temporally resolved investigation of the development of diabetic cardiomyopathy, with multiple overlaps with the HFpEF phenotype. Appearing early in disease progression are reduced skeletal muscle perfusion and exercise intolerance, both manifesting at a time when echocardiography indicates a preserved ejection fraction, early diastolic dysfunction, and mild systolic dysfunction. As skeletal muscle microvascular dysfunction from vasoconstriction worsened and dropped to nearly half of baseline value by month 10, several other HFpEF phenotypes emerged late in the study, including hypertension and late diastolic dysfunction. Classical markers, such as cardiac hypertrophy, fibrosis, and rarefaction, were not observed, likely because these are associated with hypertension and, thus, had insufficient time to develop after the late onset of hypertension.

## Data Availability Statement

The original contributions presented in the study are included in the article/supplementary material, further inquiries can be directed to the corresponding author/s.

## Ethics Statement

The animal study was reviewed and approved by Division of Comparative Medicine, University of Toronto, Protocol #20012191.

## Author Contributions

SL contributed to the study design, performing all animal procedures and imaging, data analysis, and drafting and editing of the manuscript. Y-QZ contributed to ultrasound protocol development, training, imaging, and data analysis. KV contributed to animal handling and procedures. H-LC contributed to the overall direction, conceptualization, study design, statistical analysis, and editing of the manuscript. All authors read and approved the final manuscript.

## Conflict of Interest

The authors declare that the research was conducted in the absence of any commercial or financial relationships that could be construed as a potential conflict of interest.
